# Two-dimensional photonic crystals increasing vertical light emission from Si nanocrystal-rich thin layers

**DOI:** 10.3762/bjnano.9.213

**Published:** 2018-08-24

**Authors:** Lukáš Ondič, Marian Varga, Ivan Pelant, Alexander Kromka, Karel Hruška, Robert G Elliman

**Affiliations:** 1 Institute of Physics, Czech Academy of Sciences, v.v.i., Cukrovarnická 10, 162 00, Prague 6, Czech Republic; 2 Research School of Physics and Engineering, The Australian National University, Canberra, ACT 2601, Australia

**Keywords:** leaky modes, photoluminescence, photonic crystals, silicon nanocrystals

## Abstract

We have fabricated two-dimensional photonic crystals (PhCs) on the surface of Si nanocrystal-rich SiO_2_ layers with the goal to maximize the photoluminescence extraction efficiency in the normal direction. The fabricated periodic structures consist of columns ordered into square and hexagonal pattern with lattice constants computed such that the red photoluminescence of Si nanocrystals (SiNCs) could couple to leaky modes of the PhCs and could be efficiently extracted to surrounding air. Samples having different lattice constants and heights of columns were investigated in order to find the configuration with the best performance. Spectral overlap of the leaky modes with the luminescence spectrum of SiNCs was verified experimentally by measuring photonic band diagrams of the leaky modes employing angle-resolved spectroscopy and also theoretically by computing the reflectance spectra. The extraction enhancement within different spatial angles was evaluated by means of micro-photoluminescence spectroscopy. More than 18-fold extraction enhancement was achieved for light propagating in the normal direction and up to 22% increase in overall intensity was obtained at the spatial collection angle of 14°.

## Introduction

Photonic and plasmonic nanostructures can be employed to manipulate light on the nanoscale [1–4]. For example, photons emitted within a thin waveguiding layer can be efficiently directed towards the detector by interaction with a modulation of the surface instead of being trapped inside the material [5–6]. When directionality of the extracted light is not required, a randomly patterned surface can be employed to increase the light emission intensity due to scattering [7]. On the other hand, periodic structures such as two-dimensional (2D) photonic crystals (PhCs) provide not only enhanced light extraction efficiency but also a control over the emission radiation pattern. Well-designed periodic structures can therefore be used to direct the desired wavelength into a defined direction through Bragg diffraction. Optical modes that instead of being guided in the layer can radiate into air, are referred to as leaky modes [8–9]. It was shown that by using 2D periodic patterning of group-III-nitride-based LEDs [10], similar light-extraction efficiencies as those of inverted rough-surface LEDs [11] can be obtained. The main advantage therefore remains the ability to tune the far-field radiation pattern. It is worth noting that due to reciprocity of light propagation (in the linear regime), PhC structures can also be used for light-trapping. It was shown, both theoretically [12] and experimentally [13], that by optimizing the dimensions of the PhC, very high absorption efficiencies can be achieved.

The main issue limiting the practical use of 2D PhCs is a relatively complicated and expensive fabrication process. Typically, electron beam lithography in combination with etching [14] or focused ion beam milling [15] are used to prepare photonic structures with well-defined dimensions on small areas. These approaches are therefore suitable for laboratory testings but not for practice. This issue was recently solved by developing large-scale production techniques for photonic nanostructures, such as nanoimprinting [13,16], microsphere-based lithography [17] or laser processing [18], which may open up ways towards practical applications of 2D PhCs. The latter method, for example, enabled to enhance the light extraction from InGaN/GaN quantum wells on a sapphire by 40%.

Thanks to recent progress in the understanding of light emission from silicon nanostructures, silicon nanocrystals (SiNCs) are envisaged as potential candidates for LEDs. SiNCs-based light-emitting devices with external quantum efficiencies up to 1.1% [19] and even up to 8.6% [20] have been demonstrated. Promising results have also recently been achieved for electroluminescent SiNCs embedded in SiC film [21] and in electroluminescent capacitive structures [22]. Studies that investigated possibilities to improve external quantum efficiency of such devices using photonic structures have been performed alongside.

Photonic crystal-based nanostructures were employed to enhance the electroluminescence of Si nanoclusters with a broad emission band in the near IR region [23] and also of the emission line of Er embedded in SiNCs at telecom wavelengths [24–25]. In 2013, we have shown that the light-emission from SiNCs embedded in SiO_2_ layers with the value of the peak refractive index of approx. 1.6 can be increased by structuring the smooth surface of the layer into a 2D PhC with a square lattice symmetry [26]. Recently, we have also shown that SiNC-rich layers with higher packing density than the SiNCs density of the sample studied in [26], can provide high extraction efficiencies over a wide range of the spatial angle and over a broad spectral range [27].

Here, in contrast, we will investigate a set of PhCs with various lattice constants and heights to ascertain the best performing configuration in order to maximize the light extraction from SiNC-rich layers into normal direction. We will show that by choosing a suitable density of SiNCs and dimensions of the PhCs, an up to 18-fold extraction efficiency into the direction normal to the sample plane can be obtained.

## Experimental

### Sample design and simulation

When 2D PhC structures are employed for improving the light extraction from thin layers, the extraction efficiency into the normal direction depends on the interplay of many factors, such as refractive index and thickness of the light-emitting layer, and dimensions of the PhC structure itself. In order to maximize the normal extraction, we have designed the samples such that the normally propagating leaky modes with transverse-electric (TE) and transverse-magnetic (TM) symmetry were spectrally positioned close to each other. To achieve this, the refractive index contrast between the SiNC-rich layer and the quartz substrate has to be relatively low. As it will be shown later, this leads in a real sample to spectral overlap of the two modes due to spectral broadening and enables to reach high extraction efficiencies. The low refractive-index contrast will also secure coupling of light emitted from SiNCs only into the fundamental mode with Gaussian-like energy profile and thus prevent leakage of light into higher-order modes that would be extracted into other directions. However, if the contrast in refractive index was too low the mode would be extended into the substrate and there would be insufficient overlap with the PhC on the surface of the layer. Taking this into account, suitable refractive index and dimensions of the PhCs were calculated using rigorous coupled-wave analysis (software DiffractMOD, RSoft). A compromise was found at a value of around 1.5 for the refractive index of the SiNC-rich layer. Lattice constant, diameters and heights were chosen such that the resulting leaky mode spectrum overlapped the PL spectrum of the SiNCs.

The employed software allows for the computation of transmission and reflection spectra of periodic structures as a function of the incident light polarization and the angle of incidence. As the leaky modes manifest themselves in the transmission and reflection spectra as Fano-like resonances, this approach provides a simple way to identify the leaky modes of the structure. Because the simulated leaky resonances of the studied structures were spectrally very narrow, a wavelength step of 10^−6^ nm had to be used in order to perfectly resolve their shape. Together with the asymmetric Gaussian distribution of the refractive index in the SiNC-rich layer, this required time-consuming simulations. This high spectral resolution was therefore used only for the simulation of the normal-incidence spectra, which reveals leaky modes at the Γ−point. For the case of angle-resolved reflectance simulations, which will also be presented in this paper, the spectral resolution was decreased to 10^−4^ nm. By this, even though the intensity values of the calculated resonances are not correct, their spectral position could be used for obtaining a qualitative information about the dispersion of photonic bands. As it will be shown later, the angle-resolved reflectance spectra calculated using the lower resolution are in good qualitative agreement with the measured ones.

### Fabrication of the samples

**Figure 1:**
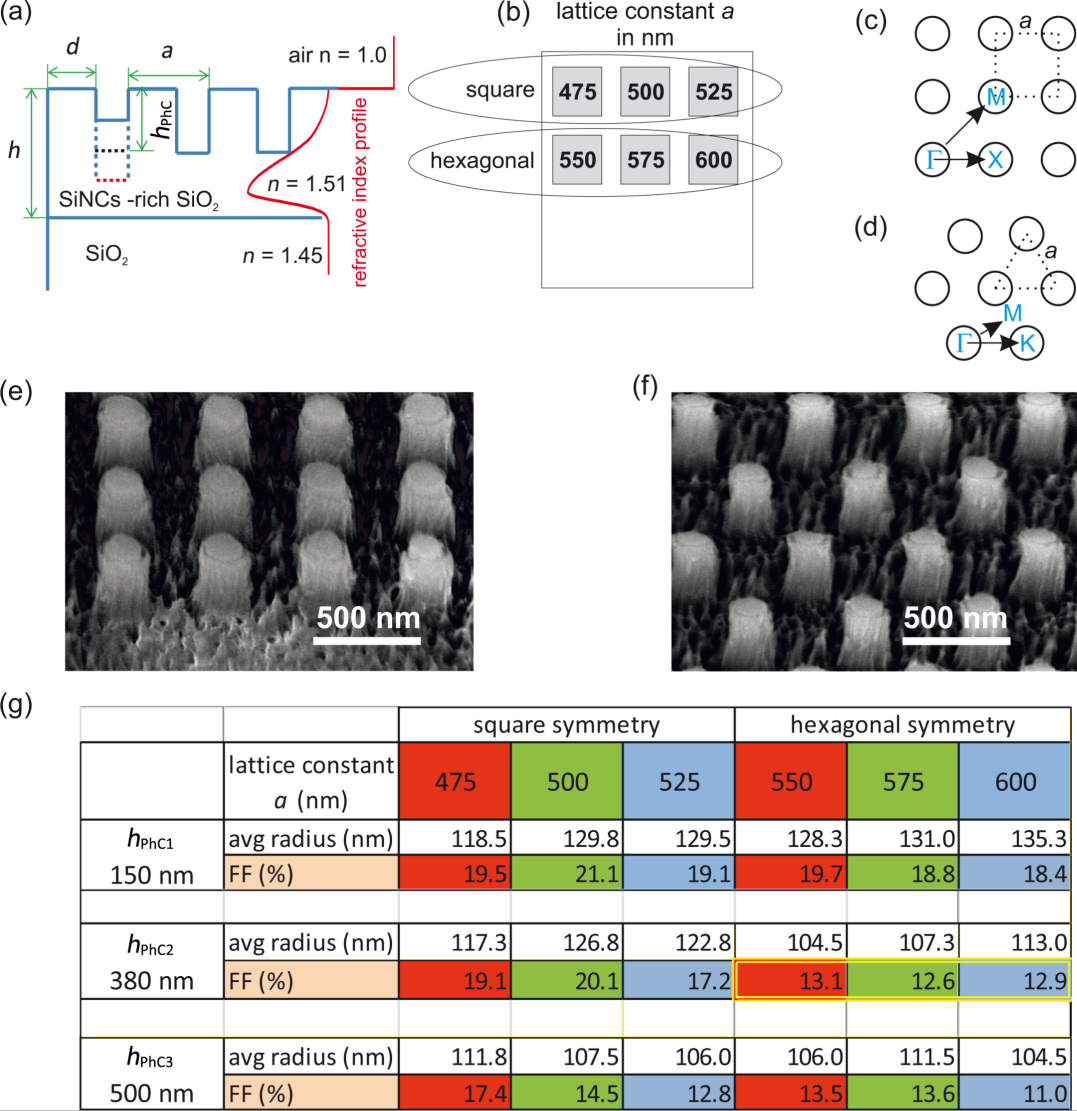
Samples under study. (a) Schematic cross section of the PhC sample. Red line shows the spatial distribution of the refractive index in the sample (not in scale). (b) Layout of the PhC samples fabricated on the SiNCs/SiO_2_ layer. Three samples with different height of the columns were prepared by increasing the etching time. Each of the three samples contains six PhC structures (the size of each is 1.3 mm × 1.3 mm) with lattice constants specified in the image in nanometers. (c, d) Schematic images of the square and hexagonal lattice symmetries with lattice constant *a*, respectively. The relevant directions of high symmetry along which the angle-resolved PL spectra were measured are also depicted. (e, f) SEM images of the square (*a* = 475 nm) and hexagonal (*a* = 550 nm) PhCs with the height *h*_PhC2_, respectively. (g) Table containing parameters of the samples: FF - filling factor.

Firstly, SiNC-rich layers were fabricated by Si ion implantation into a polished silica substrate followed by thermal annealing at 1100 °C (for details see [28]). An implant energy of 400 keV with an implant fluence of 1 × 10^17^cm^−2^ was used in order to reach the desired refractive index value. The SiNC-rich layers were ca. 800 nm thick and exhibited an asymmetric Gaussian spatial distribution of SiNCs. The final values of the refractive index within the structure were extracted from the transmission measurement by carefully fitting the Fabry–Perot resonances in the transmission spectra. The distribution of refractive index naturally follows the spatial distribution of SiNCs and therefore it can be approximated by an asymmetric Gaussian function. The spatial profile of the refractive index is schematically depicted by the red line in Figure 1a. It peaks at around 600 nm below the surface with a value of 1.51, which perfectly fulfills the requirement imposed by the simulation. We would like to note that the spatial distribution of the refractive index has already been included in the simulation discussed above.

The PhCs were fabricated as follows: The initial SiNC-rich/SiO_2_ plate was first cut into three identical pieces with the sizes of 1 cm × 2 cm. The surface of the samples was coated with 120 nm of an electron-sensitive polymer (PMMA). Then on each piece, six periodic structures (1.3 mm × 1.3 mm) were “drawn” by electron beam lithography (“e-LiNE system”, Raith GmbH, Germany) into a polymer mask forming a 2D periodic patterns of holes. A 60 nm thick gold layer was subsequently evaporated and part of the gold was removed by lift-off of the PMMA to define a mask for etching. Afterwards, the samples were etched employing capacitively coupled RF-plasma in a SF_6_ gas (Phantom LT RIE System, Trion Technology, USA) for varying periods of time (90, 240 and 320 s) in order to get samples with different heights of the PhCs. The resulting PhC structures were 2D square and hexagonal lattices of columns on the SiNC-rich layers. In total, 18 PhC structures with different parameters were fabricated.

The layout of the PhCs on one of the three SiNC-rich plates is sketched in Figure 1b. In the first row, the PhC structures possess a square lattice symmetry (Figure 1c) with three different lattice constants *a* separated by steps of 25 nm, namely *a* = 475, 500 and 525 nm. The step of 25 nm between the lattice constants allows for a systematic probing of the relatively broad PL spectrum of SiNCs with the leaky modes. In the second row, three hexagonal-lattice PhCs (Figure 1d) are designed with lattice constants of *a* = 550, 575 and 600 nm. These lattice constants are chosen in order to assure a spectral position of the leaky modes of the hexagonal PhCs that is similar to the square-lattice counterparts positioned above each of them.

As already mentioned above, each of the three SiNC-rich/SiO_2_ plates (containing always six PhCs) was etched for a given time. Depending on the sample, the heights of the columns after the etching were (i) *h*_PhC1_ ≈ 150 nm, (ii) *h*_PhC2_ ≈ 380 nm and (iii) *h*_PhC3_ ≈ 500 nm. The heights of the columns within each series of the PhC samples varies slightly (±15 nm) due to differences in the lattice constants and symmetries. A goal of this fabrication process was to prepare three sets of PhC structures with comparable properties, except the height, in order to evaluate the effect of the PhCs height on the extraction efficiency. SEM images of the final PhCs are shown in Figure 1e,f and in Figure S1 (Supporting Information File 1). Diameter of the columns varies between 220 and 270 nm depending on the etching time. Parameters of the samples with their filling factors (FF) are summarized in Figure 1g.

### Optical measurements

A custom-built automatized setup was used for the measurements of angle-resolved photoluminescence (PL) spectra. The samples were excited with a UV (325 nm) HeCd cw laser. PL spectra as a function of the extraction angle were collected by an optical fiber with low numerical aperture providing an angular resolution of less than 0.5°. The optical fiber was attached to a motorized holder rotating around the directions of high symmetry of the sample using a step of 0.5°. The output from the fiber was connected to a spectrograph coupled to a CCD camera. This specific model of the fast-gated CCD camera is spectrally sensitive only to 870 nm.

In addition, a microPL setup based on a confocal geometry was used. The same objective is employed for excitation (442 nm cw laser) of the sample and also for collection of the PL. Objectives with NA = 0.12 (5×, collection half angle of ca. 6.9°) and NA = 0.4 (20×, collection half angle of ca. 23.6°) were used. The emitted PL intensity was spectrally resolved and detected by a silicon CCD camera spectrally sensitive up to 1000 nm. In both setups, the laser was incident at a non-resonant angle, i.e. the laser is not resonantly coupled into the leaky modes of the structure. The measured PL spectra were corrected for the spectral sensitivity of the detection setup.

## Results and Discussion

### Optical fiber detection

All fabricated PhCs were first characterized by angle-resolved PL measurements in order to map the leaky modes of the structures. By this approach, only the leaky modes that overlap the SiNCs emission spectrum for different extraction angles are revealed and analyzed. The PL spectra of the PhCs are compared with the PL spectra of a reference measured under the same experimental conditions. As a reference, the partly etched planar layer surrounding the PhC structures was considered. Its thickness thus decreases with increasing the etching time and it is equal to *h*_PhC_ (see Figure 1a). As it will become clear later, comparison with the partly etched planar layer will enable to extract from measurements the influence of only PhCs on the PL emission of SiNCs.

**Figure 2:**
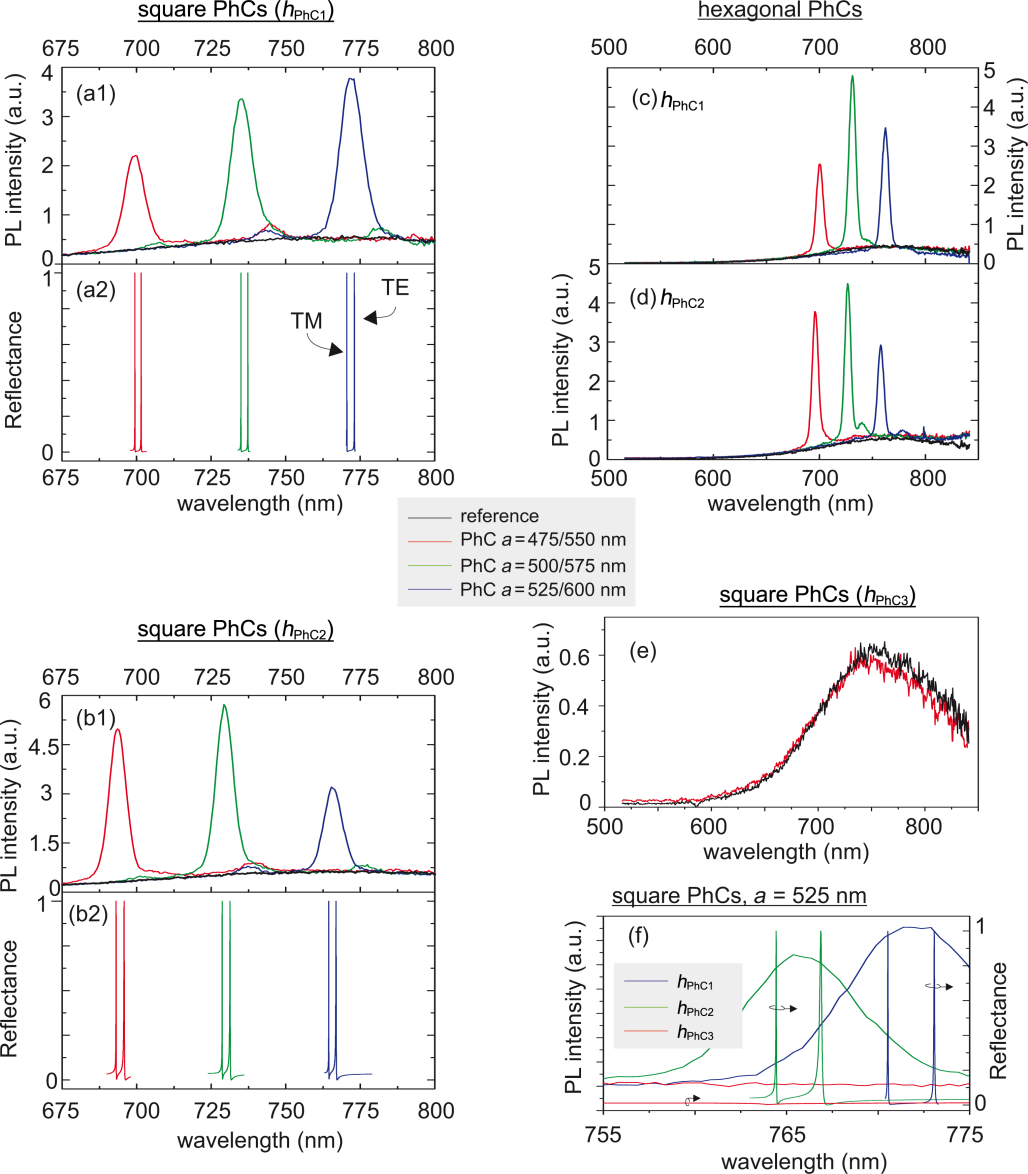
PL spectra of the PhC samples detected with an optical fiber in the direction normal to the sample plane and computed normal reflectance, which reveals the Γ-point leaky modes. The black curves represent the PL spectra of the planar reference. (a1,b1) Measured PL and (a2,b2) simulated reflectance of the square PhC with columns height of (a) *h*_PhC1_ and (b) *h*_PhC2_, respectively. (c, d) PL of the hexagonal PhC with columns height of *h*_PhC1_ and *h*_PhC2_, respectively. (e) PL of the square-lattice PhC with column height *h*_PhC3_ (red) compared to the reference (black). In panels (a–e), we use a simple self-explanatory color code in order to distinguish between the PhCs with different lattice constants. (f) PL and computed reflectance of the square-lattice PhCs with lattice constant *a* = 525 nm and column heights *h*_PhC1_ (blue), *h*_PhC2_ (green) and *h*_PhC3_ (red).

The results of these measurements for the normal (zero) extraction angle are summarized in Figure 2. Figure 2a1 and Figure 2b1 show the PL spectra of the square-lattice PhCs with the height *h*_PhC1_ and *h*_PhC2_, the lattice constant being a parameter, in comparison with the PL spectra of a reference SiNC-rich layer. The graphs are zoomed-in around the spectral part where the leaky modes, which manifest themselves as peaks superimposed on the broad PL signal from the SiNCs, occur. In parallel with the measured PL spectra, the simulated spectra of the reflectance in the direction normal to the sample plane are plotted in Figure 2a2,b2. We have obtained very good agreement of the spectral position between the measured and simulated leaky modes. The simulation shows that the measured peak in the PL spectra is composed of two leaky modes with TE and TM symmetry. The width of the measured peaks is larger than that of the simulated leaky modes mainly due to the fact that in the measurement, we are always integrating all the leaky modes extracted within the collection angle, whereas in the spectra from the simulation, only the two modes extracted exactly in the normal direction are reproduced. These graphs also clearly demonstrate the spectral tunability of the leaky modes with the lattice constant.

Figure 2c,d shows the normal PL spectra of the hexagonal-lattice PhCs with heights *h*_PhC1_ and *h*_PhC2_, respectively, the lattice constant being a parameter, in comparison with the PL spectra of the reference. In this case, the spectra are plotted within a spectral range that covers almost the whole broad PL spectrum of SiNCs. Similar to the case of the square PhCs, the PL extracted via leaky modes in the direction normal to the sample plane manifests itself as peaks superimposed on the broad PL band. Increase of the lattice constant results in a spectral red-shift of the leaky modes. By tuning the lattice constant, the peak can be shifted towards the desired wavelength.

For both PhC lattice symmetries the spectra of the PhCs and the unpatterned reference layer (the thickness of which is decreased by etching) overlap almost perfectly with respect to their intensity and shape except at the position of the leaky modes. This means that other effects such as resonant in-coupling [29] or diffraction of the excitation laser, or a difference in active surface and volume that may contribute to the increase/decrease of the PL intensity are negligible and the reason for enhanced PL intensity is purely the extraction enhancement through the leaky modes.

The above discussion is further supported by the results of the measurements performed on the PhC on which a large part of the active layer was removed by etching. Figure 2e shows that the normal PL spectrum of the extensively etched square-lattice PhC with a column height of approx. 500 nm (*h*_PhC3_) is almost equal to that of the reference SiNCs layer, which has thickness of only around 300 nm. This shows that even though the active surface of the PhC sample is larger than that of the reference layer (due to the walls of the columns), it has only negligible effect on the PL intensity. No less important is the observation that there are no leaky modes in the PL spectra of this PhC sample. This is due to the fact that the resulting structure is excessively perturbed (high columns) and can no longer support any leaky modes, an observation supported by the simulation (Figure 2f, red curve) performed using the actual dimensions taken from the SEM images. Furthermore, the structural quality of the columns is low due to the relatively long etching time employed (see SEM images in Supporting Information File 1).

Figure 2f shows a zoom-in around the leaky modes of the square-lattice PhCs (*a* = 525 nm) for the three different heights of the PhCs. An increase in the height of the columns blue-shifts the leaky modes as revealed both by the measurements and simulations, which are in perfect agreement when considering the spectral position of the leaky modes. The difference in the width of the measured and simulated resonances was discussed above. Furthermore, the simulation for the highest columns *h*_PhC3_ = 500 nm confirmed that such PhC structure does not support the existence of leaky modes at the wavelengths overlapping the emission spectrum of SiNCs.

**Figure 3:**
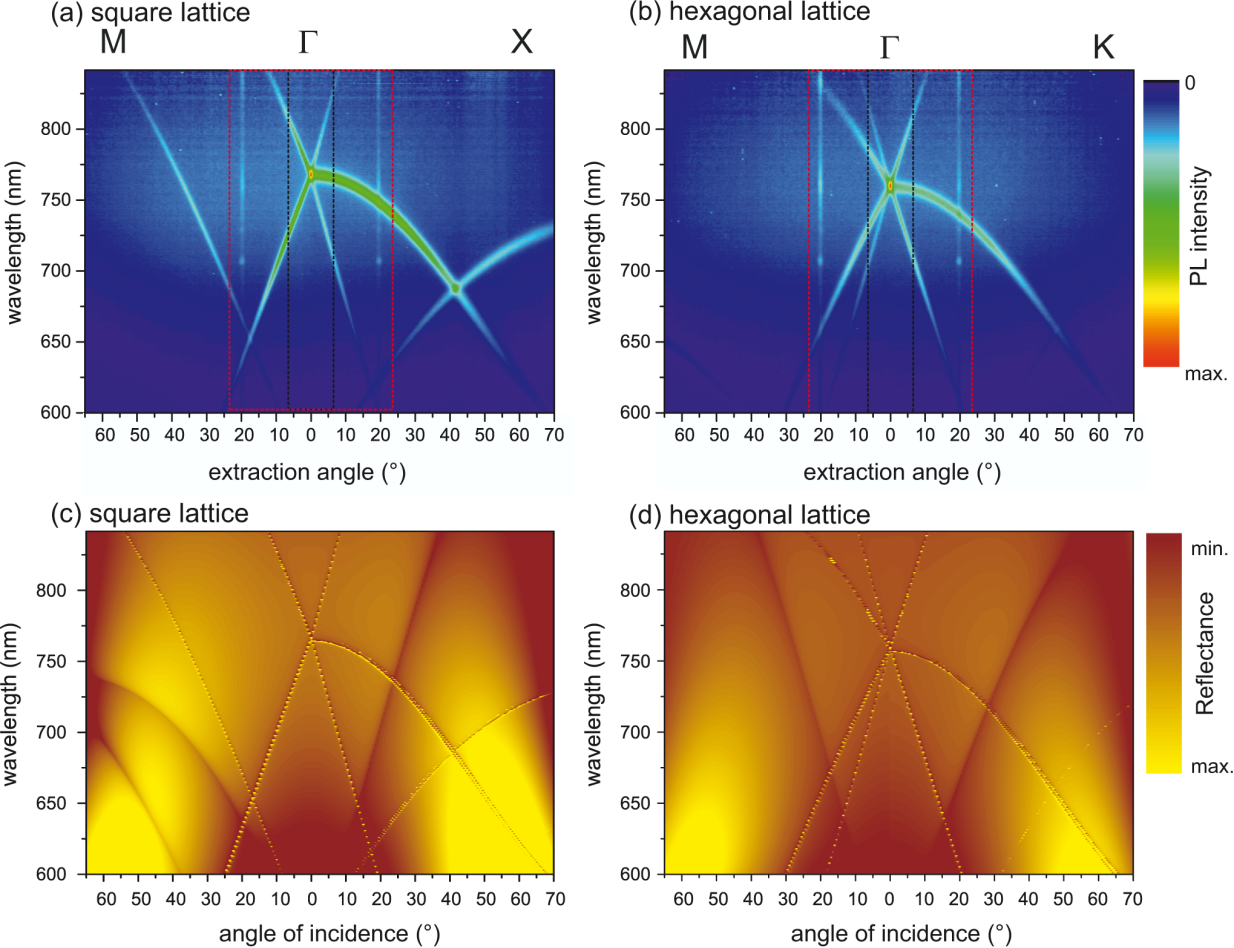
Angle-resolved PL spectra of the PhCs with *h*_PhC2_ on the SiNC-rich layer measured along the relevant directions of high symmetry. As an example, spectra of the PhCs with the “blue” lattice constants, namely (a) square lattice (*a* = 525 nm) and (b) hexagonal lattice (*a* = 600 nm) are shown. The rectangles plotted with black and red dashed lines depict the collection half angles covered with the NA = 0.12 and NA = 0.4 objectives, respectively, used in the micro-PL setup. Angle-resolved spectra of the other fabricated PhCs are given in Supporting Information File 1. Note: The vertical (intensity) lines visible in the plots at around 20° for all high-symmetry directions are artifacts of the measurement caused by the laser light entering directly into the detection fiber. (c, d) Simulated leaky modes photonic band diagrams of the square-lattice and hexagonal-lattice PhCs in panels (a) and (b), respectively. The band diagrams were obtained from simulated reflectance spectra of the PhCs.

The complete results of the angle-resolved measurements for both studied lattice symmetries but for one configuration of parameters only (height *h*PhC2, the largest “blue” lattice constants) are shown as 2D plots in Figure 3. The angle-resolved PL spectra of the other investigated PhCs are shown in Supporting Information File 1. Leaky modes spreading over the whole detected spectral range of the PL spectrum can be recognized as maxima superimposed on the broad PL signal. These measurements evidence that the leaky modes of the structure overlap the emission spectrum of the SiNCs and that the extraction is efficient even for larger detection angles. When comparing the leaky-mode structures of the square and hexagonal lattices, we see differences in the modes arising from the Γ-point along the M-point direction. In the case of the square lattice there are only two modes whereas in the case of the hexagonal lattice there are four modes. This is consistent with the higher degeneracy of the hexagonal lattice mode at the Γ-point, which means that the intensity of the mode of the hexagonal lattice at the Γ-point should be theoretically higher than that of the square lattice. Further, we can see a higher-order mode becoming visible from around 20° to higher detection angles for both directions of the square lattice. The crossing of one of these higher-order modes in the Γ–X direction with the fundamental mode is visible at around 45°.

The results of the angle-resolved PL measurements are supported by the output of our RCWA simulation. Figure 3c,d also shows the reflectance as a function of the incident angle simulated for the square-lattice and hexagonal-lattice PhCs with *a* = 525 nm and *a* = 600 nm, respectively, and *h*_PhC2_. The simulated graphs show leaky-mode band diagrams of the PhCs which agree qualitatively with the measurments. It should be noted that the dark lines in the simulated band diagrams are not leaky modes but an artifact of the simulation, which we will not discuss in detail.

### Micro-PL detection

**Figure 4:**
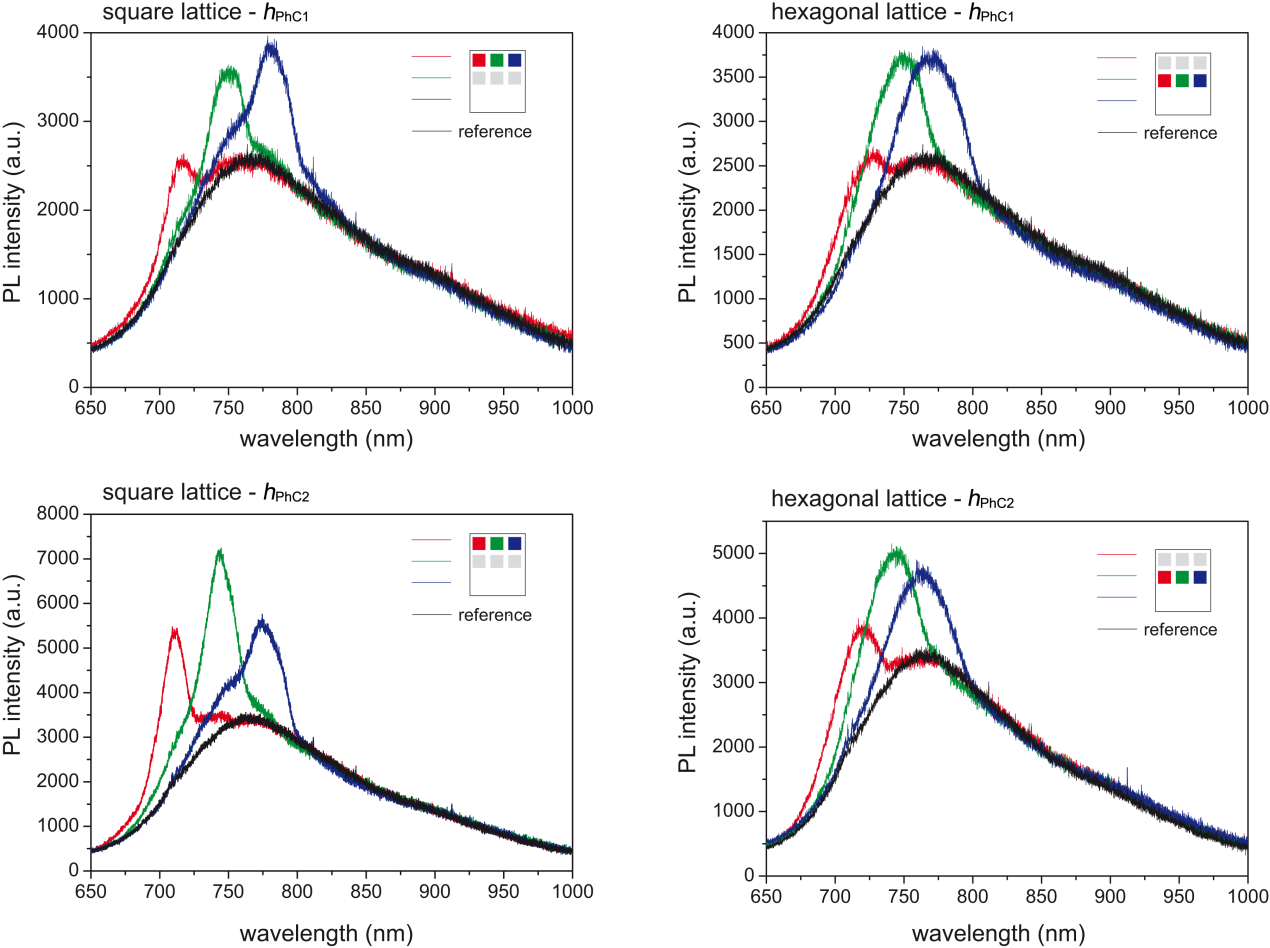
Micro-PL results for the PhCs fabricated on a SiNC-rich SiO_2_ layer collected with the objective having NA = 0.12 (collection half angle of 6.9°). PL spectra as a function of the lattice constant of the square (1st column) and hexagonal PhCs (2nd column) with column heights *h*_PhC1_ (1st row) and *h*_PhC2_ (2nd row). The color code denotes the lattice constant of the PhCs. The corresponding values can be found in Figure 1g.

Figure 4 shows PL spectra measured within a collection cone with a half angle of 6.9° of the PhC samples possessing leaky modes. PL spectra are qualitatively similar for the PhCs with heights *h*_PhC1_ and *h*_PhC2_. In all cases, a relatively broad peak is superimposed on the typical background spectrum of the SiNC-rich layer and it spectrally shifts as a function of the lattice constant. Furthermore, as expected already from the PL measurements with the optical fiber, the PhCs with the highest columns (PhC3) showed no sign of the leaky modes and we did not measure any effect of the PhC on the PL spectrum of SiNCs (not shown here).

### PL enhancement factors

**Figure 5:**
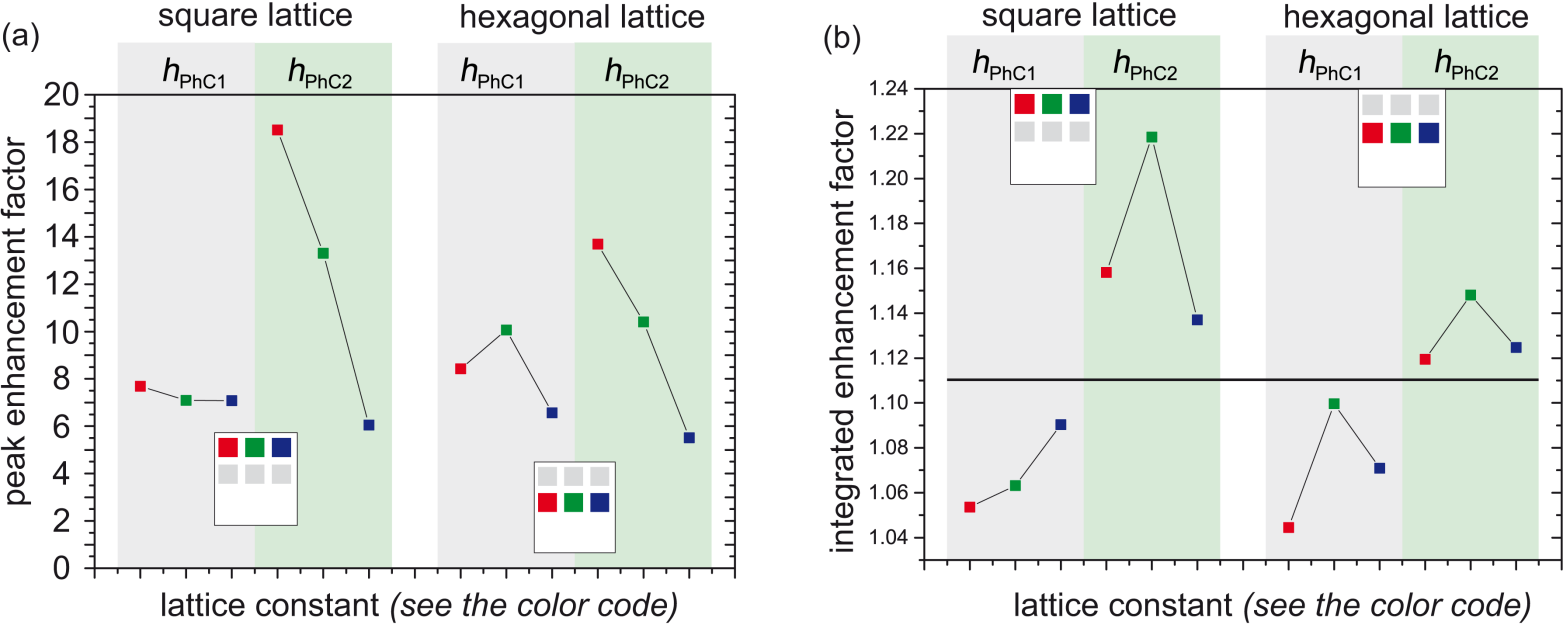
(a) The peak enhancement factor defined as a ratio of the leaky mode extracted PL intensity at the peak maximum to the PL intensity of the reference at the similar wavelength of the fabricated samples. (b) The integrated enhancement factor defined as a ratio of the area below the PL curve of the PhC to the area below the PL curve of the unpatterned reference SiNC-rich layer, both collected in a half angle of 6.9°. The horizontal line divides the results for the sample with shorter (*h*_PhC1_) and longer (*h*_PhC2_) columns.

Figure 5a shows the peak enhancement factors derived from the measurements results shown in Figure 2. We define the peak enhancement factor as a ratio of the peak intensity of the leaky mode to the PL intensity of the reference SiNC-rich layer at the same wavelength both measured with the optical fiber placed normally to the sample plane. It therefore shows how the samples perform in a very narrow collection angle for leaky modes located at and in the close vicinity of the Γ-point.

For the shorter columns (*h*_PhC1_) and both lattice symmetries (gray areas in Figure 5a), the peak enhancement factors are comparable and stay within a relatively narrow range of about 7 to 10. This follows from the fact that (i) the filling factors of these samples are comparable to each other (see Figure 1g), and (ii) the PhC structures are relatively shallow and therefore the spatial overlap of the leaky modes with the SiNCs varies only negligibly for different lattice constants. As predicted by the theory, due to the higher degeneracy of the modes at the Γ-point of the hexagonal lattice, the samples having hexagonal lattice symmetry perform slightly better for the case of the red and green lattice constants than their counterparts with square lattice symmetry. The sample with “blue” lattice constant and hexagonal lattice symmetry performs, however, slightly worse than the square PhC, most probably due to the low structural quality of the sample.

For the longer columns with *h*_PhC2_ (green areas in Figure 5a), interestingly, the peak enhancement factors obtained for the PhCs with square lattice symmetry are higher than those measured for the hexagonal symmetry PhCs, which contradicts the theoretical prediction mentioned above. The reason for this is the much lower filling factor of the samples with hexagonal symmetry being around 13% compared to the square-lattice PhCs having FF values of around 20% (Figure 1g). Even though the optimum FF depends on the specific guided mode being extracted, generally the extraction efficiency abruptly decreases when going from 20 to 10% [6,30]. Obtaining a lower FF in the case of the hexagonal-lattice PhCs with *h*_PhC2_ was unintentional and occurred due to the fact that the sample etching happens also in the lateral direction.

For the case of the PhCs with *h*_PhC2_, it also holds that for both lattice symmetries, the enhancement factor decreases with increase of the lattice constant (green areas in Figure 5a) due to the following: Lower lattice constants imply shorter wavelengths of the leaky mode at the Γ-point. This means that the mode is more confined (localized) in the active layer containing light-emitting SiNCs. And therefore, light emitted by the SiNCs can more efficiently couple into the modes with shorter wavelength, which in the end results in a higher PL intensity. This effect is more pronounced in the samples that have relatively high columns compared to the samples with relatively shallow PhC (*h*_PhC1_).

The most important observation is that the trend in the enhancement factor implies that the higher the columns of the PhC (obviously up to a reasonable extent) the better the enhancement factor. We are not taking into account the “blue” lattice PhCs with height *h*_PhC2_, which has the worse performance, since it most probably originates from their poorer structural quality.

To quantify the overall performance of the PhCs in a given solid angle with respect to the unpatterned SiNC-rich layer, we define an integrated enhancement factor as a ratio of the area below the PL curves of the PhC samples and the unpatterned reference taken in the spectral range from 650 to 1000 nm. The integrated enhancement factor is important from the point of view of utilizing this approach for LEDs based on SiNCs in which the overall intensity in a broader angle is relevant for practical uses. The integrated enhancement factors extracted from the measurements performed within the collection half angle of 6.9° for all the relevant samples are shown in Figure 5b. The highest overall intensity enhancement of about 22% was obtained for the square-lattice PhC with height *h*_PhC2_. For the PhCs with the height *h*_PhC1_, the integrated enhancement is below 10%. In general, the PhCs with longer columns perform better than the others. This holds up to a critical length above which the sample does not support the leaky modes as evidenced by the results of the PhC with *h*_PhC3_. Furthermore, in accordance with the results obtained with the optical fiber, it still holds that the PhCs with *h*_PhC2_ possessing square lattice symmetry are more efficient than the hexagonal PhCs.

PL spectra of the PhC samples measured at a broader solid angle with the objective NA = 0.4 did not show any increase in intensity compared to the reference SiNCs layer (see graphs in Supporting Information File 1). This means that the total intensity of light extracted via leaky modes into the collection half angle of ca. 23.6° is much lower compared to the total intensity of the non-perturbed PL signal radiating within the same solid angle. Collecting ranges of the two employed objectives with respect to the band diagrams are schematically shown in Figure 3. Clearly, the collection space of the objective with lower NA = 0.12 is much more densely filled with leaky modes than it is for the objective with NA = 0.4. Together with the fact that the emission is spectrally relatively broad, the effect of the PhC on the signal collected with the objective of NA = 0.4 is negligible. This means that using PhC structures for enhancing the PL in broader spatial angles of the investigated samples is not efficient and that the PhC structures are suitable when only the normal (or close to the normal) extraction is important for application. When the PL extraction at a broader spatial angle is of interest, higher implantation doses of Si ions are needed in order to obtain a higher refractive index of the active layer, which provides spectral splitting of the TE and TM modes and spectrally broader resonances [27].

## Conclusion

We have shown that the spectral position of the leaky mode extracted from a SiNC waveguide can be controlled by changing the lattice constant of a patterned PhC structure etched into the waveguide surface. More than 18-fold extraction enhancement was achieved for leaky modes extracted in the normal direction by bringing the TE and TM fundamental leaky modes spectrally close to each other. Up to 22% increase in overall intensity was obtained within the spatial collection angle of 14°, however, for the broader collection angle of 47° the extraction efficiency of the PhC sample compared to the reference was similar. This approach is therefore suitable only for enhancing normal or close to the normal PL extraction. Finally, it was found that the extraction enhancement is higher for the PhCs etched to the greater depth while maintaining a reasonably thick homogeneous SiNC-rich layer that supports the leaky modes.

## Supporting Information

File 1 Additional experimental data.
