# Supplementary material for: Two-dimensional photonic crystals increasing vertical light emission from Si nanocrystal-rich thin layers
Source: Beilstein J Nanotechnol. 2018 Aug 24;9:2287–96. doi: 10.3762/bjnano.9.213 (PMC6122158; doi:10.3762/bjnano.9.213)
Supplement: File 1 — Additional experimental data. [file Beilstein_J_Nanotechnol-09-2287-s001.pdf]

Supporting Information

for

Two-dimensional photonic crystals increasing vertical light emission from Si nanocrystal-rich thin layers

Lukáš Ondič*[1], Marian Varga[1], Ivan Pelant[1], Alexander Kromka[1], Karel Hruška[1] and Robert G. Elliman[2]

Address: [1]Institute of Physics, Czech Academy of Sciences, v.v.i., Cukrovarnická 10, 162 00, Prague 6, Czech Republic and [2]Research School of Physics and Engineering, The Australian National University, Canberra, ACT 2601, Australia

Email: Lukáš Ondič - ondic@fzu.cz

* Corresponding author

Additional experimental data

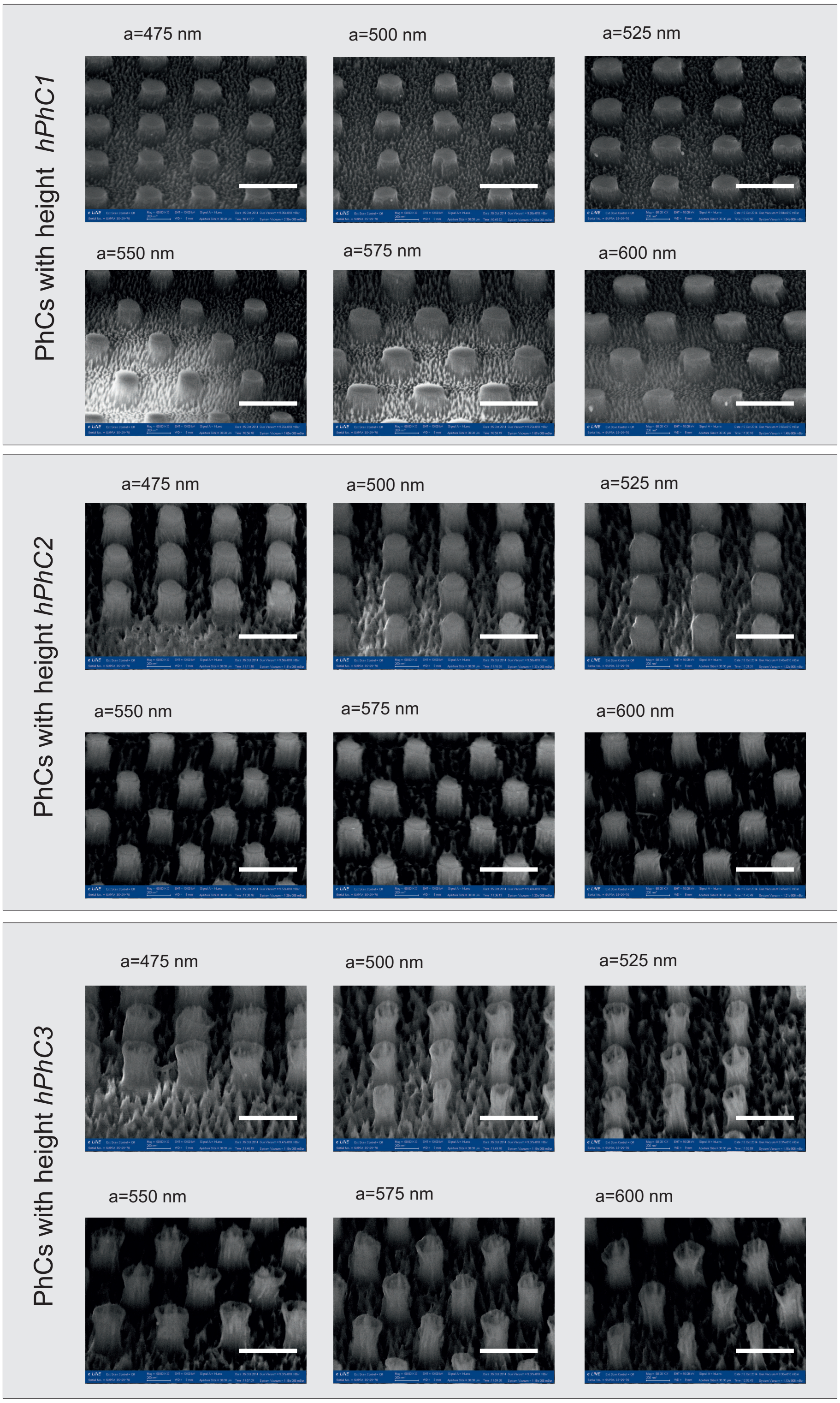

Figure S1: SEM images of the PhCs prepared on SiNCs-rich layers (implant fluence of 1×10^{17} cm^{-2}). The scale bar is 500 nm.

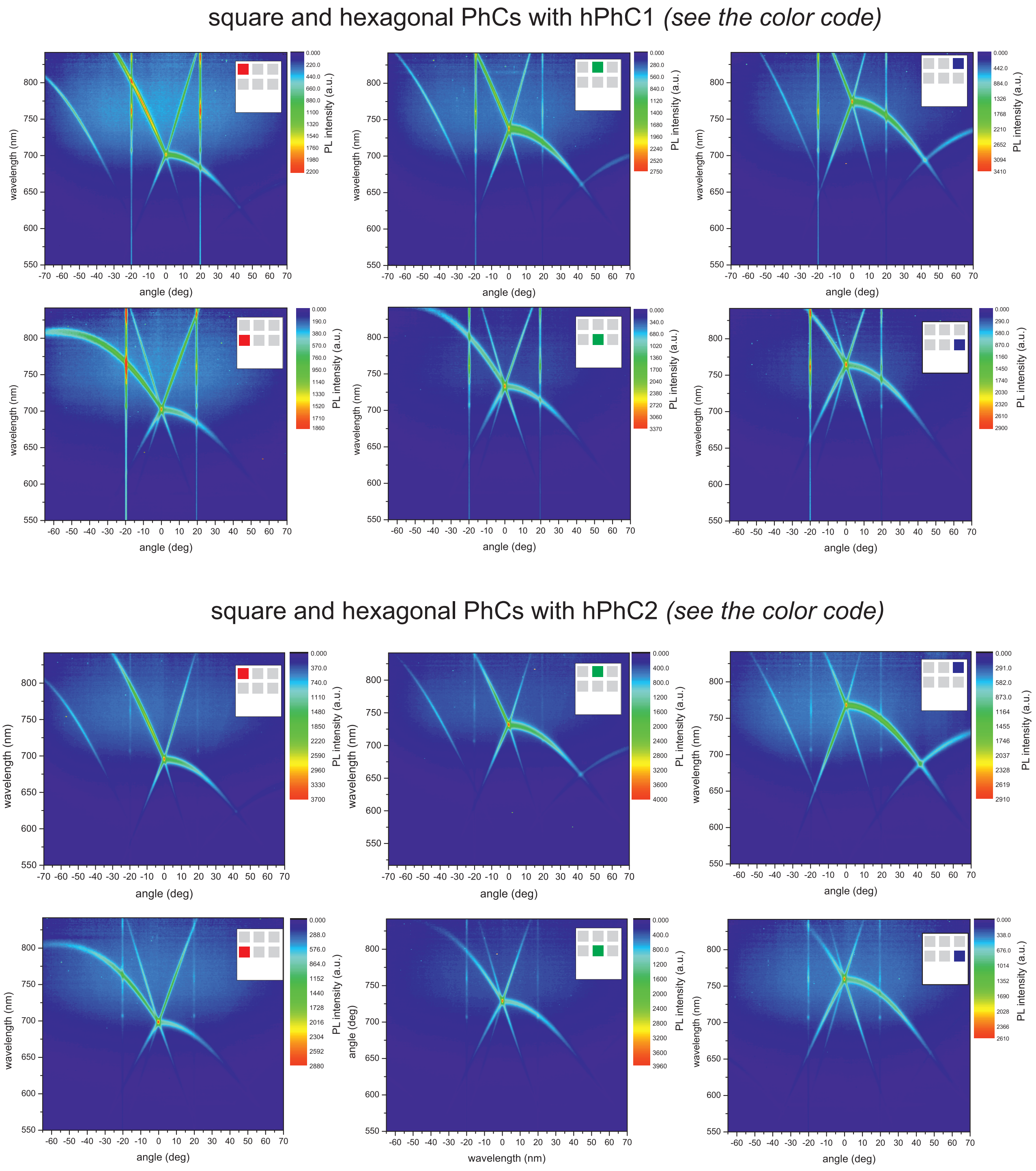

Figure S2: Angle-resolved PL measurements of the PhCs prepared on SiNCs-rich layers.

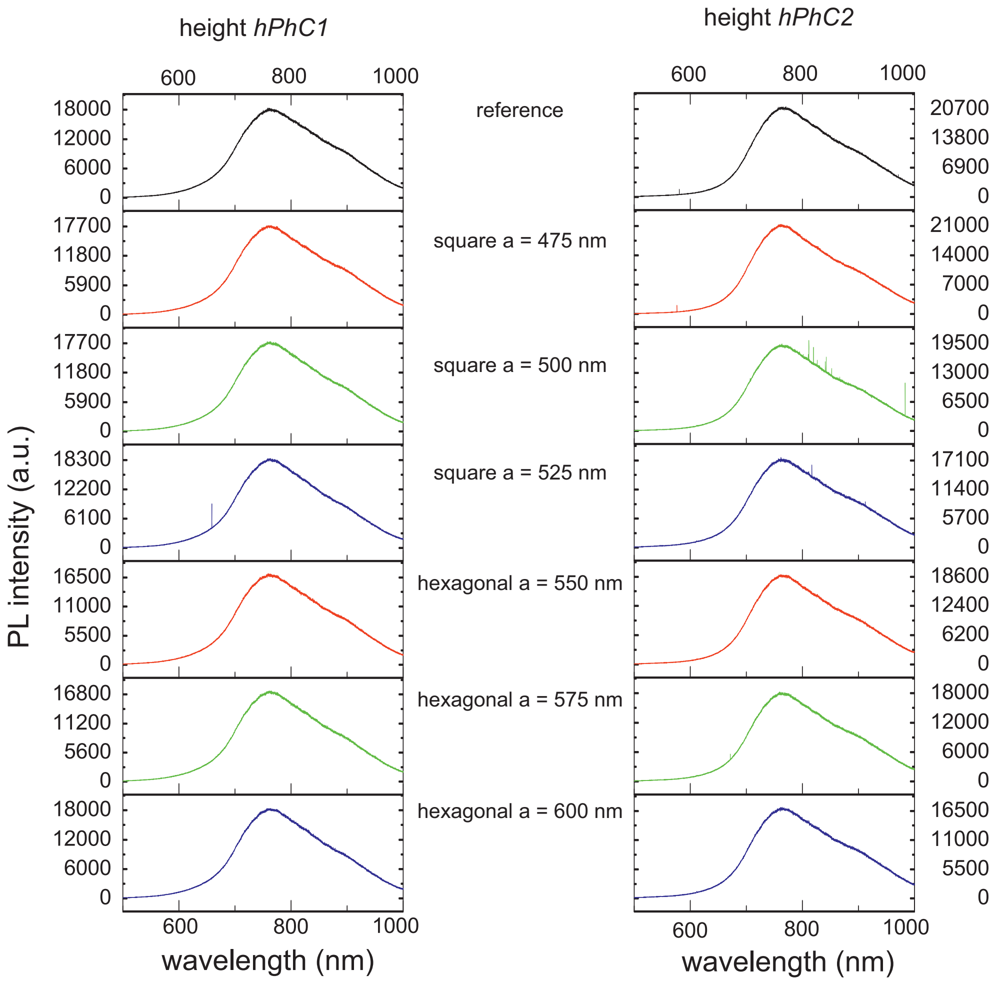

Figure S3: Micro-PL spectra measured in confocal geometry with the objective NA = 0.4 (collection half angle = 23.6°).
